# Association between affective temperaments and thalamic nuclei volumes

**DOI:** 10.3389/fpsyt.2026.1947058

**Published:** 2026-10-06

**Authors:** Hirofumi Hirakawa, Hina Kohno, Masaaki Muronaga, Toshihiko Izumi, Takuya Ogawa, Yudai Nagano, Takahiro Abeyama, Kentaro Kohno

**Affiliations:** 1 Department of Neuropsychiatry, Oita University Faculty of Medicine, Oita, Japan; 2 Health Support Center, Dannoharu Campus, Oita University, Oita, Japan

**Keywords:** brain imaging, cyclothymic temperament, magnetic resonance imaging, thalamic paratenial nucleus, thalamus

## Abstract

**Introduction:**

Affective temperaments are stable, biologically based traits that may represent subclinical manifestations or premorbid phenotypes of mood disorders. The thalamus is increasingly recognized as an active component of distributed neural networks involved in cognition, emotion, and behavioral regulation. We focused on the thalamus and conducted an exploratory analysis of the associations between affective temperaments and the volumes of thalamic nuclei in healthy participants.

**Method:**

This study included 43 healthy right-handed participants. Affective temperament was assessed using the Temperament Evaluation of Memphis, Pisa, Paris, and San Diego Autoquestionnaire. T1-weighted structural magnetic resonance imaging data were acquired using a 3T scanner and processed with FreeSurfer version 7.1.1 to obtain the estimated total intracranial volume (eTIV) and the volumes of 52 thalamic regions, comprising 25 bilateral thalamic nuclei and the bilateral whole thalamus. The primary analyses were partial Spearman correlations adjusted for age, sex, and eTIV. Given that 52 thalamic regions and five affective temperaments were examined, Bonferroni correction was applied to control the family-wise error rate for multiple comparisons. Statistical significance was defined as a Bonferroni-corrected threshold of P < 0.00019 (0.05/[52 × 5]). Additional analyses adjusted for age, sex, and ipsilateral whole thalamus volume were performed as sensitivity analyses.

**Results:**

In the primary analysis, higher cyclothymic temperament scores were significantly associated with smaller left paratenial nucleus (Pt) volume (partial ρ = -0.57, P = 0.00015, 95% CI [-0.72, -0.36]). In the sensitivity analysis adjusting for age, sex, and left whole thalamus volume, the association was attenuated (partial ρ = -0.42, P = 0.007, 95% CI [-0.62, -0.17]) and did not meet the Bonferroni-corrected significance threshold. None of the other affective temperaments were significantly associated with left Pt volume. Several other associations of comparable magnitude were observed, but none met the corrected threshold.

**Discussion:**

Higher cyclothymic temperament scores were associated with smaller left Pt volume in the primary analysis. However, the association only narrowly met the corrected significance threshold and was attenuated in the sensitivity analysis. Thus, its regional specificity and statistical robustness remain uncertain, and replication in larger independent samples is warranted.

## 1 Introduction

Affective temperaments are stable, biologically based traits that may represent subclinical manifestations or premorbid phenotypes of mood disorders such as bipolar disorder (BD) and major depressive disorder (MDD) (1). Akiskal et al., identifies five distinct affective temperaments: depressive, cyclothymic, hyperthymic, irritable, and anxious (2). These temperaments are presumed to possess a robust biological basis, manifesting early in life and persisting throughout the lifespan, and are linked to genetic factors (1). With regard to affective psychopathology, cyclothymic, hyperthymic, and irritable temperaments have been associated with BD, whereas depressive and anxious temperaments have been associated with MDD (3).

We have previously investigated the associations between affective temperaments and structural and functional neuroimaging findings in healthy adults. Cyclothymic temperament has been associated with greater gray matter volume in the left medial frontal gyrus, white matter microstructure in the corpus callosum, corona radiata, and external capsule, as well as function in the left lingual gyrus and right superior parietal lobule (4–7). Hyperthymic temperament has been associated with greater gray matter volume in the left medial frontal gyrus and with white matter microstructural differences involving the corpus callosum and left superior longitudinal fasciculus (4, 5). Functional imaging studies have also suggested an association between hyperthymic temperament and activity in the left inferior orbitofrontal cortex, particularly during light-related preference and decision-making tasks (8, 9). More recently, irritable temperament was associated with glucose metabolism in the left insula and right cerebellum (10). To further investigate the neural substrates associated with affective temperament, we focused on the thalamus in the present study. The thalamus is increasingly recognized as an active component of distributed neural networks involved in cognition, emotion, and behavioral regulation (11, 12). These functional roles suggest that structural variations in the thalamus may be related to individual differences in affective temperament. However, to the best of our knowledge, no previous study has investigated these associations. Recent advances in neuroimaging have enabled the segmentation of thalamic nuclei (13). Accordingly, we conducted an exploratory analysis to examine associations between affective temperaments and the volumes of thalamic nuclei in healthy participants.

## 2 Methods

### 2.1 Participants

This study analyzed data from 44 healthy Japanese participants derived from one previously published study and one unpublished study. Twenty healthy participants were enrolled before a light-exposure intervention (bright or dim light) in our previous study (14), which was approved by the Institutional Review Board of the Oita University Faculty of Medicine (B15-019). An additional 24 healthy participants were included from an unpublished study that aimed to investigate the associations between affective temperament and neuroimaging findings in patients with mood disorders and healthy participants. However, this study was discontinued following the departure of the principal investigator and remained unpublished. The study protocol was approved by the Institutional Review Board of the Oita University Faculty of Medicine (B13-031). All data were collected at Oita University. The eligibility criteria for participants included the absence of any current or past psychiatric disorders, as determined by the Mini-International Neuropsychiatric Interview, being 20 years of age or older, and right-handed. The exclusion criteria encompassed serious physical diseases, including neurological disorders, previous head trauma or brain lesions, pregnancy, and lactation. Of the 44 participants, 43 underwent magnetic resonance imaging (MRI) using the MAGNETOM Verio, whereas one participant was scanned using the MAGNETOM Skyra Fit. To ensure consistency in scanner type and acquisition conditions, we excluded the single participant scanned with the Skyra Fit and restricted the analysis to the 43 participants scanned with the Verio. All other eligible participants were retained in the study, with no exclusions due to unusable imaging data. There was no overlap among participants. The methodology adhered to ethical standards and pertinent national and institutional guidelines for human experimentation, as well as the principles of the 1975 Helsinki Declaration, as revised in 2024. To utilize brain imaging data from previous studies for analysis, the research proposal was made available on our department’s website, allowing participants to decline consent for the use of their prior data. This opt-out study was approved by the Institutional Review Board of the Oita University Faculty of Medicine on August 4th, 2023 (approval number: 2599).

### 2.2 Assessment of affective temperament and mood symptoms

The Temperament Evaluation of Memphis, Pisa, Paris, and San Diego Autoquestionnaire (TEMPS-A) consists of 110 questions answered with either “yes” or “no” and assesses five affective temperaments: depressive, cyclothymic, hyperthymic, irritable, and anxious. The number of “yes” responses for each temperament dimension was used in all primary statistical analyses. For descriptive comparison with the non-clinical Japanese participants reported by Matsumoto et al. (15), we additionally transformed the TEMPS-A scores according to their scoring method, in which “No” was scored as 1 and “Yes” as 2, and the total score for each temperament was divided by the number of corresponding items. The scale was originally developed by Akiskal et al., the scale has demonstrated adequate reliability and validity in both international and Japanese populations (2, 15). In the present study, scores for all five affective temperaments were used. Mood symptoms were assessed in all participants using the 17-item Hamilton Depression Rating Scale (HAM-D) and the Young Mania Rating Scale (YMRS). The TEMPS-A, HAM-D, and YMRS assessments were conducted on the same day as the MRI scan.

### 2.3 MRI acquisition and image processing

MRI data were acquired using 3-T MRI scanners (MAGNETOM Verio; Siemens, Erlangen, Germany), each equipped with a maximum gradient strength of 45 mT/m and a 32-channel head coil. T1-weighted structural images were obtained using a three-dimensional magnetization-prepared rapid gradient-echo (MPRAGE) sequence in the sagittal plane with the following parameters: repetition time (TR) = 2040 ms, echo time (TE) = 2.53 ms, inversion time (TI) = 900 ms, flip angle = 9°, field of view (FOV) = 192 mm, and voxel size =1×1×1 mm³. T1-weighted images were processed using FreeSurfer version 7.1.1 running on Ubuntu 18.04. Standard cortical reconstruction and subcortical segmentation were performed using the automated recon-all pipeline with default settings. This pipeline includes removal of non-brain tissue, intensity normalization, Talairach transformation, and automated cortical and subcortical segmentation. The estimated total intracranial volume (eTIV) was obtained from the FreeSurfer segmentation outputs (16). Thalamic nuclei were subsequently segmented using the FreeSurfer thalamic nuclei segmentation tool based on the probabilistic atlas which combines ex vivo MRI and histological data (13). In this process, volumes of 52 thalamic regions, comprising 25 bilateral thalamic nuclei and the bilateral whole thalamus were calculated. All T1-weighted images and FreeSurfer segmentation outputs were visually inspected using FreeView. Images were assessed for gross motion or acquisition artifacts, skull-stripping or reconstruction failures, and obvious segmentation errors. The thalamic nuclei were also visually inspected for gross anatomical misplacement. No manual editing of the thalamic nuclei labels was performed.

### 2.4 Statistical analysis

First, the distributions of affective temperament scores were assessed using the Kolmogorov-Smirnov test. For temperament scores that were not normally distributed, rank-based analyses were used. Affective temperament scores, thalamic nucleus volumes, age, eTIV, and whole thalamus volume were converted to ranks. The primary analyses were partial Spearman correlation analyses examining associations between ranked affective temperament scores and ranked thalamic nucleus volumes, with adjustment for ranked age, sex, and ranked eTIV. Given that 52 thalamic regions and five affective temperaments were examined, Bonferroni correction was applied to control the family-wise error rate within this primary family of 260 tests. Accordingly, statistical significance for the primary analyses was defined as P < 0.00019 (0.05/[52 × 5]). The 95% confidence intervals for partial Spearman correlation coefficients were estimated using a nonparametric bootstrap procedure with 1,000 resamples and the percentile method implemented in SPSS. Furthermore, as a sensitivity analysis, additional partial Spearman correlation analyses were performed to examine whether associations with individual thalamic nuclei might reflect broader variation in hemispheric thalamic volume. Partial correlations between ranked affective temperament scores and ranked thalamic nucleus volumes were calculated with adjustment for ranked age, sex, and ranked ipsilateral whole thalamus volume. These sensitivity analyses were considered supportive and were not used to determine statistical significance in the primary analyses. In addition, although the FreeSurfer thalamic nuclei segmentation framework has demonstrated high overall test-retest reliability, with reported intraclass correlation coefficients exceeding 0.85, paratenial nucleus (Pt) reliability data have not been reported (13). Therefore, the across-subject Pearson correlation between left and right Pt volumes was additionally calculated as an indirect proxy for measurement reliability. The present study was a secondary analysis of existing data from two previously conducted studies and was not prospectively designed to test the current hypothesis. Accordingly, an *a priori* sample size calculation was not performed. All statistical analyses were performed using SPSS version 32.0.

## 3 Results

This study included 43 healthy adults. The mean age of the participants was 26.1 ± 6.3 years, and 23 were male. HAM-D and YMRS scores were within normal limits, indicating the absence of clinically significant mood symptoms. The Kolmogorov-Smirnov test revealed that none of the five affective temperament scores were normally distributed (**Table 1**; **Supplementary Table 1**; **Supplementary Figure 1**). A comparison of affective temperament scores between the present study and the non-clinical Japanese participants reported by Matsumoto et al. (15) is presented in **Supplementary Table 2**. Demographic characteristics, affective temperament scores, eTIV, and the volumes of the bilateral thalamic nuclei and whole thalamus are summarized in **Table 1**.

**Table 1:** The demographic characteristics of the participants.

Variable	Participants (n=43)
Age mean (s.d., range)	26.1 (6.3, 20-55)
Sex (Male, Female)	23, 20
HAM-D mean (s.d., range)	0.3 (0.7, 0-3)
YMRS mean (s.d., range)	0.05 (0.2, 0-1)
The scores of TEMPS-A	
Depressive temperament scores, mean (s.d., range)	5.4 (2.7, 1-11)
Cyclothymic temperament scores, mean (s.d., range)	3.2 (3.1, 0-10)
Hyperthymic temperament scores, mean (s.d., range)	4.3 (4.3, 0-15)
Irritable temperament scores, mean (s.d., range)	1.8 (2.0, 0-7)
Anxious temperament scores, mean (s.d., range)	2.9 (3.1, 0-12)
Neuroimaging characteristics	
Estimated total intracranial volume, mm^3^ mean (s.d., range)	1544964.4 (164198.6, 1231973.0-1954192.0)
Thalamic nucleus	
Left LGN volume, mm^3^ mean (s.d., range)	268.7 (36.0, 190.6-357.1)
Right LGN volume, mm^3^ mean (s.d., range)	317.6 (39.1, 206.4-406.9)
Left MGN volume, mm^3^ mean (s.d., range)	106.8 (20.4, 63.5-152.9)
Right MGN volume, mm^3^ mean (s.d., range)	119.2 (20.6, 73.0-152.8)
Left Pul volume, mm^3^ mean (s.d., range)	238.4 (28.8, 181.6-305.6)
Right Pul volume, mm^3^ mean (s.d., range)	306.9 (47.6, 207.9-429.4)
Left PuM volume, mm^3^ mean (s.d., range)	1140.4 (122.1, 876.5-1407.3)
Right PuM volume, mm^3^ mean (s.d., range)	1366.5 (159.8, 1053.8-1723.5)
Left L-Sg volume, mm^3^ mean (s.d., range)	24.1 (7.8, 10.4-44.9)
Right L-Sg volume, mm^3^ mean (s.d., range)	20.6 (5.5, 10.9-32.9)
Left VPL volume, mm^3^ mean (s.d., range)	887.0 (94.5, 684.5-1193.0)
Right VPL volume, mm^3^ mean (s.d., range)	1047.7 (134.1, 833.2-1332.0)
Left CM volume, mm^3^ mean (s.d., range)	238.6 (35.3, 174.4-350.6)
Right CM volume, mm^3^ mean (s.d., range)	254.5 (36.4, 181.6-324.3)
Left VLa volume, mm^3^ mean (s.d., range)	643.8 (64.2, 491.9-809.3)
Right VLa volume, mm^3^ mean (s.d., range)	676.9 (66.5, 569.0-850.5)
Left PuA volume, mm^3^ mean (s.d., range)	234.8 (23.4, 199.7-320.1)
Right PuA volume, mm^3^ mean (s.d., range)	269.7 (28.2, 217.2-360.9)
Left MDm volume, mm^3^ mean (s.d., range)	820.2 (84.2, 652.1-950.2)
Right MDm volume, mm^3^ mean (s.d., range)	866.7 (86.0, 657.1-1024.7)
Left Pf volume, mm^3^ mean (s.d., range)	57.7 (10.5, 31.1-80.6)
Right Pf volume, mm^3^ mean (s.d., range)	65.0 (13.8, 27.1-87.7)
Left VAmc volume, mm^3^ mean (s.d., range)	32.7 (3.4, 26.2-39.3)
Right VAmc volume, mm^3^ mean (s.d., range)	34.7 (3.7, 28.6-42.9)
Left MDl volume, mm^3^ mean (s.d., range)	282.5 (30.0, 224.7-335.6)
Right MDl volume, mm^3^ mean (s.d., range)	299.7 (29.7, 242.1-368.9)
Left CeM volume, mm^3^ mean (s.d., range)	67.5 (8.6, 53.2-91.6)
Right CeM volume, mm^3^ mean (s.d., range)	72.7 (10.2, 57.8-95.0)
Left VA volume, mm^3^ mean (s.d., range)	437.3 (39.5, 343.2-511.0)
Right VA volume, mm^3^ mean (s.d., range)	423.9 (40.0, 353.8-527.3)
Left MV(Re) volume, mm^3^ mean (s.d., range)	13.0 (1.9, 9.3-17.5)
Right MV(Re) volume, mm^3^ mean (s.d., range)	14.7 (2.5, 9.4-20.7)
Left VM volume, mm^3^ mean (s.d., range)	22.9 (4.2, 15.1-38.3)
Right VM volume, mm^3^ mean (s.d., range)	29.3 (4.8, 22.0-38.1)
Left CL volume, mm^3^ mean (s.d., range)	33.6 (6.6, 22.2-47.0)
Right CL volume, mm^3^ mean (s.d., range)	36.6 (7.6, 21.2-58.1)
Left PuL volume, mm^3^ mean (s.d., range)	182.9 (31.1, 133.8-321.4)
Right PuL volume, mm^3^ mean (s.d., range)	220.7 (37.9, 160.9-313.5)
Left Pt volume, mm^3^ mean (s.d., range)	6.6 (0.8, 4.5-9.3)
Right Pt volume, mm^3^ mean (s.d., range)	7.9 (0.9, 5.8-9.5)
Left AV volume, mm^3^ mean (s.d., range)	132.9 (18.9, 93.6-169.8)
Right AV volume, mm^3^ mean (s.d., range)	145.7 (21.9, 101.4-193.7)
Left Pc volume, mm^3^ mean (s.d., range)	4.0 (0.5, 3.1-4.8)
Right Pc volume, mm^3^ mean (s.d., range)	4.7 (0.5, 3.5-5.8)
Left VLp volume, mm^3^ mean (s.d., range)	837.6 (77.0, 688.2-1064.5)
Right VLp volume, mm^3^ mean (s.d., range)	893.3 (92.3, 744.6-1131.6)
Left LP volume, mm^3^ mean (s.d., range)	131.5 (22.5, 98.3-188.8)
Right LP volume, mm^3^ mean (s.d., range)	128.1 (20.9, 85.5-174.7)
Left LD volume, mm^3^ mean (s.d., range)	28.3 (7.5, 17.5-42.7)
Right LD volume, mm^3^ mean (s.d., range)	28.3 (7.7, 12.8-41.6)
Left whole thalamus volume, mm^3^ mean (s.d., range)	6873.8 (600.5, 5655.1-8397.2)
Right whole thalamus volume, mm^3^ mean (s.d., range)	7651.7 (705.7, 6261.2-9285.2)

AV, Anteroventral; CeM, Central medial; CM, Centromedian; CL, Central lateral; HAM-D, Hamilton Depression Rating Scale; LD, Laterodorsal; LGN, Lateral geniculate; LP, Lateral posterior; L-SG, Limitans (suprageniculate); MGN, Medial geniculate; MDl, Mediodorsal lateral parvocellular; MDm, Mediodorsal medial magnocellular; Pc, Paracentral; Pf, Parafascicular; Pt, Paratenial; PuA, Pulvinar anterior; PuI, Pulvinar inferior; PuL, Pulvinar lateral; PuM, Pulvinar medial; MV-Re, Reuniens (medial ventral); s.d., Standard deviation; TEMPS-A, Temperament Evaluation of Memphis, Pisa, Paris, and San Diego Autoquestionnaire; VA, Ventral anterior; VAmc, Ventral anterior magnocellular; VLa, Ventral lateral anterior; VLp, Ventral lateral posterior; VM, Ventromedial; VPL, Ventral posterolateral; YMRS, Young Mania Rating Scale.

In the primary analysis, higher cyclothymic temperament scores were significantly associated with smaller left Pt volume after adjustment for age, sex, and eTIV (partial ρ = -0.57, P = 0.00015, 95% CI [-0.72, -0.36]) (**Figure 1**; **Table 2**). In the sensitivity analysis adjusting for age, sex, and left whole-thalamus volume, the association was attenuated (partial ρ = -0.42, P = 0.007, 95% CI [-0.62, -0.17]) and did not meet the Bonferroni-corrected significance threshold (**Table 2**). A representative example of left Pt identification based on FreeSurfer segmentation is shown in **Supplementary Figure 2**. Left and right Pt volumes were significantly positively correlated across participants (Pearson’s r = 0.58, P = 0.00005, 95% CI [0.34, 0.75]). Several additional associations of comparable magnitude were observed between other affective temperaments and thalamic nuclei; however, none met the Bonferroni-corrected significance threshold (**Supplementary Tables 3**, **4**).

**Figure 1:**
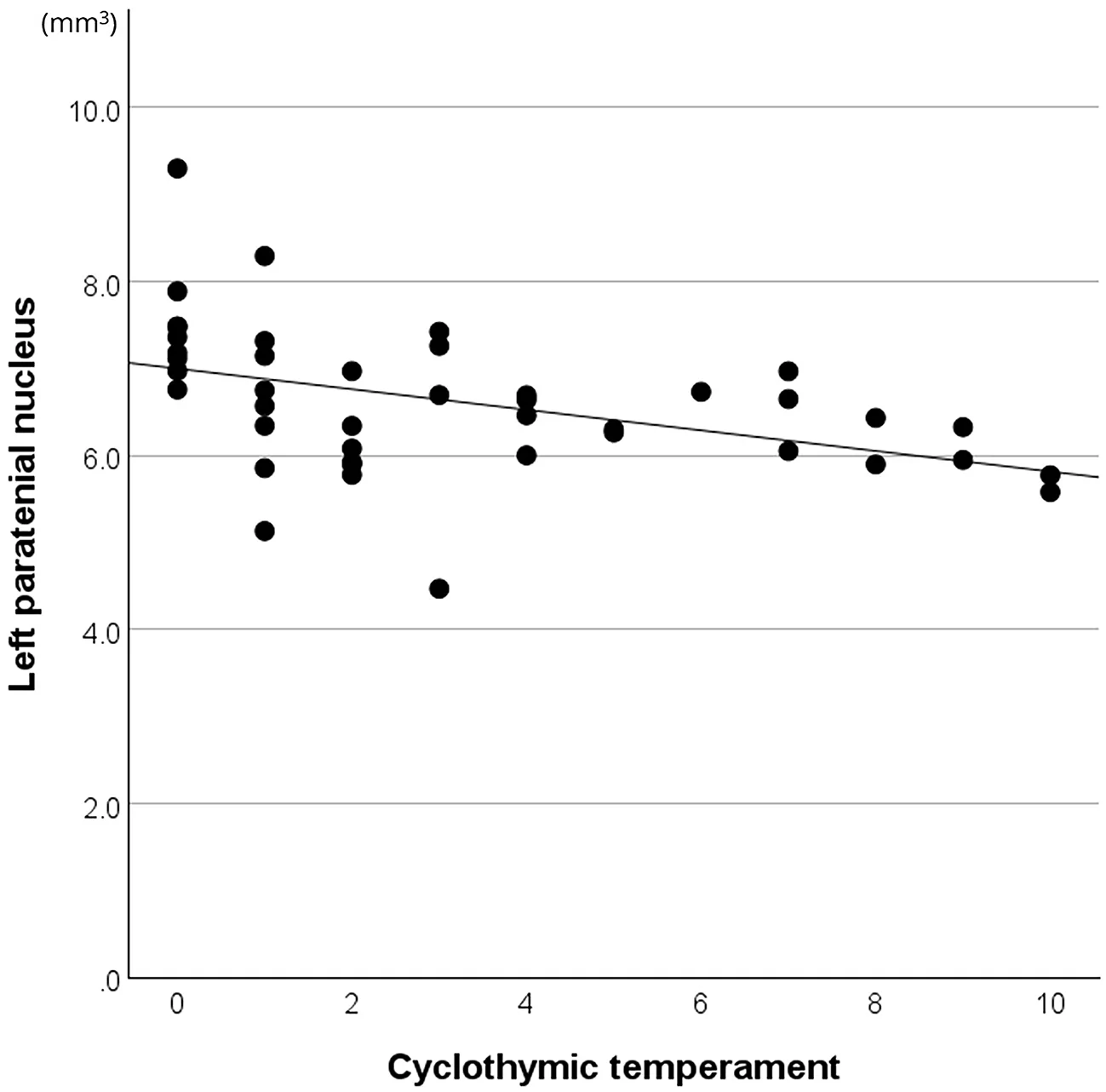
Correlations between cyclothymic temperament and left thalamic paratenial nucleus. A significant negative association between cyclothymic temperament scores and the left thalamic paratenial nucleus volume after adjustment for age, sex, and eTIV (partial ρ = -0.57, P = 0.00015, 95% CI [-0.72, -0.36]).

**Table 2:** Correlations between affective temperament and left thalamic paratenial nucleus volumes.

Variable	Left Pt, Partial correlation adjusted for age, sex, and eTIV (The primary analysis)	Left Pt, Partial correlation adjusted for age, sex, and ipsilateral whole thalamus volume. (The sensitivity analysis)
Depressive temperament	partial ρ = -0.13, p = 0.42, 95% CI [-0.46, 0.2]	partial ρ = -0.18, p = 0.26, 95% CI [-0.56, 0.2]
Cyclothymic temperament	**partial ρ = -0.57, p = 0.00015,** **95% CI [-0.72, -0.36]**	partial ρ = -0.42, p = 0.007, 95% CI [-0.62, -0.17]
Hyperthymic temperament	partial ρ = -0.23, p = 0.16, 95% CI [-0.49, 0.07]	partial ρ = 0.02, p = 0.9, 95% CI [-0.27, 0.33]
Irritable temperament	partial ρ = -0.37, p = 0.02, 95% CI [-0.62, -0.08]	partial ρ = -0.1, p = 0.56, 95% CI [-0.44, 0.22]
Anxious temperament	partial ρ = -0.2, p = 0.22, 95% CI [-0.46, 0.05]	partial ρ = -0.18, p = 0.26, 95% CI [-0.54, 0.13]

Estimated total intracranial volume: eTIV, Paratenial: Pt.

Statistical significance was defined as a Bonferroni-corrected threshold of P < 0.00019 in the primary analysis. Boldface indicates statistically significant results.

## 4 Discussion

In the present study, higher cyclothymic temperament scores were associated with smaller left Pt volume in healthy participants after adjustment for age, sex, and eTIV; however, it was attenuated after adjustment for age, sex, and left whole thalamus volume. Several associations of comparable magnitude were also observed for other temperament dimensions and thalamic nuclei, although they did not meet the Bonferroni-corrected significance threshold. To the best of our knowledge, this is the first study to report an association between affective temperaments and thalamic nuclei volumes.

The Pt is components of the dorsal midline thalamus (17). Although the functional role of the Pt is less well-defined, anatomical studies have shown that the Pt project to the limbic and prefrontal regions involved in affective and motivational regulation, including the medial prefrontal cortex, nucleus accumbens, bed nucleus of the stria terminalis, and amygdala (18). More broadly, the midline thalamic nuclei are thought to contribute to arousal, reward processing, and motivated behavior, internal state monitoring, and the integration of limbic information (19–21). Regarding affective temperaments, the TEMPS-A scores of the participants in the present study were broadly comparable from those previously reported by Matsumoto et al. (15). In addition, the HAM-D and YMRS scores indicated the absence of clinically relevant mood symptoms, suggesting that our participants represented a non-clinical population with minimal mood symptoms. Cyclothymic temperament is characterized by marked fluctuations in self-esteem, alternating periods of overconfidence and low self-confidence, variations in professional and creative productivity, and shifts between unexplained tearfulness and joyfulness (2). These characteristics reflect instability not only in mood but also in self-appraisal, energy, reward sensitivity, and goal-directed behaviors.

Although the present study included only a non-clinical population with minimal mood symptoms, the association between cyclothymic temperament and left Pt volume was observed. However, because the relative strengths of the associations across temperament dimensions, thalamic nuclei, and hemispheres were not formally compared, this finding should not be interpreted as evidence that the association is specific to cyclothymic temperament or confined to the left Pt. Furthermore, the association between cyclothymic temperament and left Pt volume was attenuated from a partial correlation coefficient of -0.57 after adjustment for age, sex, and eTIV to -0.42 after adjustment for age, sex, and left whole thalamus volume. However, the association between cyclothymic temperament and left whole thalamus volume itself did not meet the corrected significance threshold. In addition, because the Pt is contained within the left thalamus, adjustment for left whole thalamus volume necessarily removes variance shared between Pt volume and whole thalamus volume. Therefore, the present analysis cannot determine whether the observed association reflects a regionally localized relationship involving the left Pt or a more global relationship involving the left thalamus, and the present findings do not establish regional specificity to the left Pt. Moreover, the association in the primary analysis only narrowly met the Bonferroni-corrected significance threshold (P = 0.00015 vs. P < 0.00019) and did not remain significant in the sensitivity analysis adjusting for ipsilateral whole thalamus volume. Thus, the statistical robustness of this finding is limited, and it should be interpreted cautiously and replicated in larger independent samples.

This study has several limitations. First, the sample size was relatively small, which may have limited the statistical power. Second, the cross-sectional design precludes causal inference, preventing the determination of whether a smaller Pt volume precedes the development of cyclothymic traits or arises in association with them. Third, large-scale brain-wide association studies have demonstrated that associations between inter-individual differences in MRI-derived measures and complex behavioral or mental health phenotypes are generally smaller and less reproducible than suggested by studies with modest sample sizes, with structural MRI measures and self-reported mental health phenotypes being particularly susceptible to effect-size inflation (22). Moreover, large case-control studies of bipolar disorder have reported relatively small standardized differences in whole thalamus volume (23, 24). Although the Spearman correlation coefficient observed in the present study cannot be directly compared with case-control Cohen’s d estimates because these effect-size metrics arise from different study designs and statistical frameworks, the relatively large magnitude of the observed association in our small sample warrants caution regarding sampling variability and potential effect-size inflation. Accordingly, the present finding should be regarded as a preliminary effect-size estimate requiring independent replication in substantially larger samples, rather than as evidence that variation in cyclothymic temperament produces a greater neuroanatomical effect than bipolar disorder itself. Fourth, the Pt is a very small anatomical structure with a volume of only several cubic millimeters, making its segmentation inherently susceptible to partial volume effects, limitations in image resolution, and minor inaccuracies in anatomical registration or label assignment. Although the FreeSurfer thalamic nuclei segmentation framework has demonstrated high overall test-retest reliability, with reported intraclass correlation coefficients exceeding 0.85, Pt-specific reliability data have not been reported (13). In the present sample, left and right Pt volumes were moderately correlated across participants, providing an indirect indication of bilateral consistency in Pt volume estimates. However, this cross-sectional bilateral correlation is not equivalent to a direct test–retest reliability estimate and therefore does not establish the reliability of Pt segmentation. In addition, despite visual inspection of all segmentation outputs, the small size and limited anatomical contrast of the Pt make accurate validation by visual inspection difficult. Therefore, Pt volume measurements should be interpreted with caution, and their reliability. Fifth, the functional role of the Pt remains less well defined. Although animal neuroanatomical studies have implicated medial thalamic nuclei in limbic and motivational processes, the extent to which these findings can be extrapolated to the human Pt remains uncertain. Because the present study assessed structural measures only, we did not directly investigate functional aspects such as affective salience, reward processing, motivational drive, or functional connectivity. Therefore, future studies using functional MRI, diffusion imaging, and connectivity analyses are needed to establish the functional relevance of the observed association with left Pt volume. Sixth, although bipolar disorder and bipolar spectrum disorders are considered to have a substantial genetic component, the present study did not assess a family history of mood disorders. Seventh, several associations of comparable magnitude were observed across affective temperament dimensions and thalamic nuclei, although they did not meet the Bonferroni-corrected significance threshold. Because the relative strengths of these associations were not formally compared, the present findings do not establish specificity to cyclothymic temperament or the left Pt. Larger independent studies are needed to determine the robustness and regional distribution of these associations. Finally, as the participants were healthy right-handed adults, the generalizability of the results to individuals with bipolar disorder, other psychiatric disorders, left-handed individuals, or more diverse populations is uncertain.

In conclusion, higher cyclothymic temperament scores were associated with smaller left Pt volume in healthy participants. However, its regional specificity and statistical robustness remain uncertain, and replication in larger independent samples is warranted.

## Data Availability

The raw data supporting the conclusions of this article will be made available by the authors, without undue reservation.
