# Supplementary material for: Association between affective temperaments and thalamic nuclei volumes
Source: Front Psychiatry. 2026 Oct 6;17:1947058. doi: 10.3389/fpsyt.2026.1947058 (PMC13680398; doi:10.3389/fpsyt.2026.1947058)
Supplement: Supplementary file 1 [file SupplementaryFile1.docx]

Supplementary Material

# 1 Supplementary Tables

**Table S1. The distributions of affective temperament scores.**

| Variable | Kolmogorov-Smirnov statistic | p value |
| --- | --- | --- |
| Depressive temperament | 0.135 | 0.047 |
| Cyclothymic temperament | 0.189 | 0.0005 |
| Hyperthymic temperament | 0.158 | 0.009 |
| Irritable temperament | 0.218 | 0.00002 |
| Anxious temperament | 0.221 | 0.00001 |

**Table S2.** **Comparison of affective temperament scores between the present study and the non-clinical Japanese participants.**

Abbreviation; The Temperament Evaluation of Memphis, Pisa, Paris, and San Diego Autoquestionnaire: TEMPS-A

| The scores of TEMPS-A using the methods of Matsumoto et al, 2005 [15] | Present study | Matsumoto et al, 2005 [15] |
| --- | --- | --- |
| Depressive temperament scores, mean (s.d.) | 1.26 (0.13) | 1.34 (0.18) |
| Cyclothymic temperament scores, mean (s.d.) | 1.15 (0.15) | 1.21 (0.21) |
| Hyperthymic temperament scores, mean (s.d.) | 1.21 (0.21) | 1.27 (0.20) |
| Irritable temperament scores, mean (s.d.) | 1.09 (0.1) | 1.14 (0.16) |
| Anxious temperament scores, mean (s.d.) | 1.11 (0.12) | 1.19 (0.20) |

**Table S3. Associations between affective temperament scores and thalamic nucleus volumes assessed using partial Spearman’s rank correlation coefficients adjusted** **for ranked age, sex, and ranked eTIV.**

Abbreviation; Anteroventral: AV, Central medial: CeM, Centromedian: CM, Central lateral: CL, Laterodorsal: LD, Lateral geniculate: LGN, Lateral posterior: LP, Limitans (suprageniculate): L-SG, Medial geniculate: MGN, Mediodorsal lateral parvocellular: MDl, Mediodorsal medial magnocellular: MDm, Paracentral: Pc, Parafascicular: Pf, Paratenial: Pt, Pulvinar anterior: PuA, Pulvinar inferior: PuI, Pulvinar lateral: PuL, Pulvinar medial: PuM, Reuniens (medial ventral): MV-Re, Ventral anterior: VA, Ventral anterior magnocellular: VAmc, Ventral lateral anterior: VLa, Ventral lateral posterior: VLp, Ventromedial: VM, Ventral posterolateral: VPL

|  | Depressive | Cyclothymic | Hyperthymic | Irritable | Anxious |
| --- | --- | --- | --- | --- | --- |
| Left LGN | partial ρ = 0.03, p = 0.85, 95% CI [-0.26, 0.33] | partial ρ = -0.15, p = 0.37,  95% CI [-0.43, 0.15] | partial ρ = 0.03, p = 0.87,  95% CI [-0.25, 0.32] | partial ρ = -0.13, p = 0.44,  95% CI [-0.4, 0.16] | partial ρ = -0.09, p = 0.56,  95% CI [-0.39, 0.2] |
| Right LGN | partial ρ = 0.07, p = 0.67  95% CI [-0.28, 0.41] | partial ρ = 0.04, p = 0.83,  95% CI [-0.3, 0.33] | partial ρ = -0.01, p = 0.94,  95% CI [-0.36, 0.31] | partial ρ = 0.18, p = 0.26,  95% CI [-0.14, 0.45] | partial ρ = 0.15, p = 0.37,  95% CI [-0.16, 0.41] |
| Left MGN | partial ρ = 0.12, p = 0.45, 95% CI [-0.18, 0.37] | partial ρ = -0.06, p = 0.7,  95% CI [-0.38, 0.25] | partial ρ = -0.28, p = 0.08,  95% CI [-0.52, -0.04] | partial ρ = -0.11, p = 0.5,  95% CI [-0.4, 0.23] | partial ρ = 0.13, p = 0.43,  95% CI [-0.2, 0.42] |
| Right MGN | partial ρ = 0.09, p = 0.57, 95% CI [-0.23, 0.44] | partial ρ = -0.01, p = 0.93,  95% CI [-0.36, 0.31] | partial ρ = -0.32, p = 0.04,  95% CI [-0.6, -0.05] | partial ρ = -0.02, p = 0.92,  95% CI [-0.35, 0.33] | partial ρ = 0.12, p = 0.45,  95% CI [-0.19, 0.42] |
| Left Pul | partial ρ = -0.2, p = 0.23,  95% CI [-0.49, 0.11] | partial ρ = -0.23, p = 0.15,  95% CI [-0.49, 0.04] | partial ρ = -0.13, p = 0.42,  95% CI [-0.41, 0.21] | partial ρ = -0.15, p = 0.35,  95% CI [-0.46, 0.19] | partial ρ = -0.26, p = 0.11,  95% CI [-0.55, 0.06] |
| Right Pul | partial ρ= -0.08, p = 0.64, 95% CI [-0.35, 0.27] | partial ρ = 0.24, p = 0.13,  95% CI [-0.03, 0.5] | partial ρ = 0.09, p = 0.58,  95% CI [-0.22, 0.37] | partial ρ = 0.16, p = 0.33,  95% CI [-0.16, 0.46] | partial ρ = -0.06, p = 0.72,  95% CI [-0.34, 0.24] |
| Left PuM | partial ρ = -0.22, p = 0.17, 95% CI [-0.51, 0.07] | partial ρ = -0.26, p = 0.1,  95% CI [-0.48, -0.02] | partial ρ = -0.37, p = 0.02,  95% CI [-0.6, -0.07] | partial ρ = -0.42, p = 0.008,  95% CI [-0.65, -0.15] | partial ρ = -0.26, p = 0.11,  95% CI [-0.53, 0.01] |
| Right PuM | partial ρ = -0.12, p = 0.45, 95% CI [-0.38, 0.16] | partial ρ = 0.07, p = 0.69,  95% CI [-0.25, 0.34] | partial ρ = -0.03, p = 0.83,  95% CI [-0.39, 0.26] | partial ρ = 0.04, p = 0.83,  95% CI [-0.34, 0.41] | partial ρ = -0.1, p = 0.55,  95% CI [-0.41, 0.19] |
| Left L-Sg | partial ρ = -0.16, p = 0.33, 95% CI [-0.44, 0.12] | partial ρ = -0.35, p = 0.026,  95% CI [-0.6, -0.03] | partial ρ = -0.4, p = 0.01,  95% CI [-0.65, -0.12] | partial ρ = -0.17, p = 0.3,  95% CI [-0.45, 0.15] | partial ρ = 0.07, p = 0.67,  95% CI [-0.22, 0.37] |
| Right L-Sg | partial ρ = -0.11, p = 0.5, 95% CI [-0.45, 0.23] | partial ρ = -0.11, p = 0.5,  95% CI [-0.42, 0.18] | partial ρ = -0.51, p = 0.0008,  95% CI [-0.79, -0.23] | partial ρ = -0.06, p = 0.72,  95% CI [-0.37, 0.27] | partial ρ = 0.14, p = 0.39,  95% CI [-0.2, 0.45] |
| Left VPL | partial ρ = 0.17, p = 0.31,  95% CI [-0.11, 0.4] | partial ρ = -0.27, p = 0.1,  95% CI [-0.53, 0.03] | partial ρ = 0.007, p = 0.97,  95% CI [-0.29, 0.32] | partial ρ = -0.26, p = 0.11,  95% CI [-0.53, 0.03] | partial ρ = -0.04, p = 0.82,  95% CI [-0.32, 0.23] |
| Right VPL | partial ρ = 0.11, p = 0.52,  95% CI [-0.15, 0.38] | partial ρ = -0.03, p = 0.83,  95% CI [-0.39, 0.27] | partial ρ = -0.07, p = 0.67,  95% CI [-0.35, 0.25] | partial ρ = -0.006, p = 0.97,  95% CI [-0.38, 0.34] | partial ρ = 0.08, p = 0.64,  95% CI [-0.2, 0.33] |
| Left CM | partial ρ = 0.05, p = 0.76, 95% CI [-0.21, 0.3] | partial ρ = -0.29, p = 0.07,  95% CI [-0.54, 0.002] | partial ρ = -0.13, p = 0.43,  95% CI [-0.37, 0.16] | partial ρ = -0.21, p = 0.19,  95% CI [-0.49, 0.14] | partial ρ = 0.007, p = 0.96,  95% CI [-0.27, 0.27] |
| Right CM | partial ρ = 0.05, p = 0.76,  95% CI [-0.27, 0.38] | partial ρ = -0.17, p = 0.28,  95% CI [-0.47, 0.14] | partial ρ = -0.12, p = 0.46,  95% CI [-0.4, 0.17] | partial ρ = -0.07, p = 0.67,  95% CI [-0.39, 0.28] | partial ρ = 0.08, p = 0.61,  95% CI [-0.2, 0.37] |
| Left VLa | partial ρ = -0.04, p = 0.81,  95% CI [-0.39, 0.3] | partial ρ = -0.27, p = 0.1,  95% CI [-0.52, 0.04] | partial ρ = -0.25, p = 0.12,  95% CI [-0.51, 0.05] | partial ρ = -0.03, p = 0.86,  95% CI [-0.38, 0.31] | partial ρ = 0.14, p = 0.39,  95% CI [-0.22, 0.48] |
| Right VLa | partial ρ = 0.02, p = 0.91, 95% CI [-0.3, 0.35] | partial ρ = -0.08, p = 0.61,  95% CI [-0.42, 0.24] | partial ρ = -0.17, p = 0.31,  95% CI [-0.49, 0.14] | partial ρ = -0.19, p = 0.25,  95% CI [-0.61, 0.19] | partial ρ = -0.03, p = 0.88,  95% CI [-0.3, 0.23] |
| Left PuA | partial ρ = 0.09, p = 0.6,  95% CI [-0.25, 0.37] | partial ρ = -0.29, p = 0.08,  95% CI [-0.51, -0.03] | partial ρ = -0.55, p = 0.00025,  95% CI [-0.72, -0.31] | partial ρ = -0.38, p = 0.02,  95% CI [-0.62, -0.11] | partial ρ = -0.01, p = 0.93,  95% CI [-0.31, 0.27] |
| Right PuA | partial ρ = -0.005, p = 0.98, 95% CI [-0.34, 0.31] | partial ρ = 0.12, p = 0.48,  95% CI [-0.22, 0.39] | partial ρ = -0.13, p = 0.43,  95% CI [-0.45, 0.22] | partial ρ = -0.05, p = 0.77,  95% CI [-0.44, 0.28] | partial ρ = 0.03, p = 0.86,  95% CI [-0.26, 0.34] |
| Left MDm | partial ρ = 0.04, p = 0.79,  95% CI [-0.28, 0.38] | partial ρ = -0.33, p = 0.04,  95% CI [-0.6, 0.03] | partial ρ = -0.32, p = 0.04,  95% CI [-0.61, -0.03] | partial ρ = -0.4, p = 0.01,  95% CI [-0.59, -0.14] | partial ρ = -0.14, p = 0.38,  95% CI [-0.41, 0.18] |
| Right MDm | partial ρ = 0.04, p = 0.83, 95% CI [-0.29, 0.43] | partial ρ = -0.01, p = 0.93,  95% CI [-0.32, 0.29] | partial ρ = -0.28, p = 0.08,  95% CI [-0.57, 0.06] | partial ρ = -0.09, p = 0.59,  95% CI [-0.41, 0.24] | partial ρ = -0.16, p = 0.33,  95% CI [-0.43, 0.14] |
| Left Pf | partial ρ = -0.09, p = 0.58, 95% CI [-0.36, 0.17] | partial ρ = -0.39, p = 0.01,  95% CI [-0.63, -0.11] | partial ρ = -0.16, p = 0.32,  95% CI [-0.45, 0.16] | partial ρ = -0.18, p = 0.27,  95% CI [-0.48, 0.16] | partial ρ = -0.05, p = 0.78,  95% CI [-0.36, 0.23] |
| Right Pf | partial ρ = 0.03, p = 0.85, 95% CI [-0.3, 0.34] | partial ρ = -0.45, p = 0.003,  95% CI [-0.67, -0.22] | partial ρ = -0.36, p = 0.02,  95% CI [-0.64, -0.08] | partial ρ = -0.33, p = 0.04,  95% CI [-0.56, -0.07] | partial ρ = -0.002, p = 0.99,  95% CI [-0.24, 0.24] |
| Left VAmc | partial ρ = -0.08, p = 0.63, 95% CI [-0.38, 0.26] | partial ρ = -0.29, p = 0.07,  95% CI [-0.54, -0.001] | partial ρ = -0.24, p = 0.13,  95% CI [-0.52, 0.08] | partial ρ = -0.06, p = 0.72,  95% CI [-0.37, 0.26] | partial ρ = -0.03, p = 0.84,  95% CI [-0.39, 0.3] |
| Right VAmc | partial ρ = -0.15, p = 0.36, 95% CI [-0.5, 0.23] | partial ρ = -0.1, p = 0.54,  95% CI [-0.44, 0.19] | partial ρ = -0.2, p = 0.21,  95% CI [-0.52, 0.14] | partial ρ = -0.16, p = 0.32,  95% CI [-0.58, 0.19] | partial ρ = -0.09, p = 0.56,  95% CI [-0.39, 0.19] |
| Left MDl | partial ρ = -0.04, p = 0.8, 95% CI [-0.38, 0.31] | partial ρ = -0.38, p = 0.01,  95% CI [-0.61, -0.04] | partial ρ = -0.41, p = 0.008,  95% CI [-0.68, -0.12] | partial ρ = -0.44, p = 0.005,  95% CI [-0.65, -0.17] | partial ρ = -0.09, p = 0.59,  95% CI [-0.38, 0.26] |
| Right MDl | partial ρ = 0.11, p = 0.51, 95% CI [-0.19, 0.42] | partial ρ = 0.02, p = 0.92,  95% CI [-0.31, 0.34] | partial ρ = -0.25, p = 0.12,  95% CI [-0.52, 0.01] | partial ρ = -0.14, p = 0.39,  95% CI [-0.44, 0.18] | partial ρ = 0.06, p = 0.69,  95% CI [-0.2, 0.34] |
| Left CeM | partial ρ = -0.15, p = 0.37, 95% CI [-0.43, 0.18] | partial ρ = -0.05, p = 0.77,  95% CI [-0.33, 0.25] | partial ρ = -0.09, p = 0.58,  95% CI [-0.37, 0.29] | partial ρ = 0.003, p = 0.99,  95% CI [-0.36, 0.36] | partial ρ = -0.04, p = 0.82,  95% CI [-0.37, 0.33] |
| Right CeM | partial ρ = -0.18, p = 0.26, 95% CI [-0.43, 0.15] | partial ρ = -0.25, p = 0.12,  95% CI [-0.54, 0.03] | partial ρ = -0.25, p = 0.12,  95% CI [-0.53, 0.08] | partial ρ = -0.29, p = 0.07,  95% CI [-0.6, 0.04] | partial ρ = -0.29, p = 0.07,  95% CI [-0.51, -0.05] |
| Left VA | partial ρ = -0.04, p = 0.83, 95% CI [-0.35, 0.31] | partial ρ = -0.2, p = 0.21,  95% CI [-0.5, 0.11] | partial ρ = -0.14, p = 0.4,  95% CI [-0.43, 0.16] | partial ρ = 0.02, p = 0.9,  95% CI [-0.3, 0.35] | partial ρ = 0.08, p = 0.62,  95% CI [-0.24, 0.43] |
| Right VA | partial ρ = -0.13, p = 0.44, 95% CI [-0.45, 0.23] | partial ρ = 0.004, p = 0.98,  95% CI [-0.33, 0.3] | partial ρ = -0.31, p = 0.049,  95% CI [-0.55, -0.01] | partial ρ = -0.21, p = 0.2,  95% CI [-0.53, 0.13] | partial ρ = -0.07, p = 0.69,  95% CI [-0.34, 0.21] |
| Left MV(Re) | partial ρ = -0.28, p = 0.08, 95% CI [-0.58, 0.08] | partial ρ = -0.23, p = 0.16,  95% CI [-0.49, 0.07] | partial ρ = -0.12, p = 0.46,  95% CI [-0.43, 0.23] | partial ρ = 0.03, p = 0.85,  95% CI [-0.26, 0.32] | partial ρ = -0.16, p = 0.31,  95% CI [-0.48, 0.16] |
| Right MV(Re) | partial ρ = -0.21, p = 0.19, 95% CI [-0.52, 0.14] | partial ρ = -0.1, p = 0.53,  95% CI [-0.38, 0.18] | partial ρ = -0.15, p = 0.35,  95% CI [-0.45, 0.18] | partial ρ = -0.14, p = 0.38,  95% CI [-0.46, 0.17] | partial ρ = -0.25, p = 0.12,  95% CI [-0.52, 0.03] |
| Left VM | partial ρ = -0.06, p = 0.71, 95% CI [-0.37, 0.24] | partial ρ = -0.28, p = 0.08,  95% CI [-0.54, 0.03] | partial ρ = -0.14, p = 0.4,  95% CI [-0.4, 0.14] | partial ρ = -0.14, p = 0.41,  95% CI [-0.45, 0.2] | partial ρ = -0.004, p = 0.98,  95% CI [-0.31, 0.26] |
| Right VM | partial ρ = -0.03, p = 0.88, 95% CI [-0.3, 0.24] | partial ρ = -0.05, p = 0.77,  95% CI [-0.38, 0.31] | partial ρ = -0.11, p = 0.5,  95% CI [-0.44, 0.21] | partial ρ = -0.15, p = 0.35,  95% CI [-0.49, 0.18] | partial ρ = -0.04, p = 0.8,  95% CI [-0.25, 0.32] |
| Left CL | partial ρ = -0.18, p = 0.27, 95% CI [-0.41, 0.07] | partial ρ = -0.16, p = 0.33,  95% CI [-0.46, 0.16] | partial ρ = 0.29, p = 0.07,  95% CI [0.03, 0.52] | partial ρ = -0.12, p = 0.45,  95% CI [-0.46, 0.19] | partial ρ = -0.36, p = 0.02,  95% CI [-0.64, -0.05] |
| Right CL | partial ρ = -0.15, p = 0.36, 95% CI [-0.43, 0.14] | partial ρ = -0.18, p = 0.26,  95% CI [-0.46, 0.13] | partial ρ = 0.07, p = 0.69,  95% CI [-0.23, 0.38] | partial ρ = -0.11, p = 0.49,  95% CI [-0.39, 0.19] | partial ρ = -0.23, p = 0.16,  95% CI [-0.51, 0.11] |
| Left PuL | partial ρ = 0.25, p = 0.12, 95% CI [-0.04, 0.48] | partial ρ = -0.02, p = 0.92,  95% CI [-0.25, 0.26] | partial ρ = -0.33, p = 0.04,  95% CI [-0.55, -0.04] | partial ρ = -0.2, p = 0.21,  95% CI [-0.51, 0.15] | partial ρ = 0.2, p = 0.21,  95% CI [-0.09, 0.48] |
| Right PuL | partial ρ = 0.26, p = 0.11, 95% CI [-0.05, 0.59] | partial ρ = 0.42, p = 0.008,  95% CI [0.1, 0.68] | partial ρ = 0.12, p = 0.47,  95% CI [-0.24, 0.45] | partial ρ = 0.19, p = 0.24,  95% CI [-0.14, 0.51] | partial ρ = 0.34, p = 0.03,  95% CI [0.06, 0.6] |
| Left Pt | partial ρ = -0.13, p = 0.42, 95% CI [-0.46, 0.2] | **partial ρ = -0.57, p = 0.00015,  95% CI [-0.72, -0.36]** | partial ρ = -0.23, p = 0.16,  95% CI [-0.49, 0.07] | partial ρ = -0.37, p = 0.02,  95% CI [-0.62, -0.08] | partial ρ = -0.2, p = 0.22,  95% CI [-0.46, 0.05] |
| Right Pt | partial ρ = 0.003, p = 0.98, 95% CI [-0.29, 0.29] | partial ρ = -0.08, p = 0.65,  95% CI [-0.41, 0.21] | partial ρ = -0.18, p = 0.26,  95% CI [-0.47, 0.13] | partial ρ = 0.00007, p = 1,  95% CI [-0.39, 0.38] | partial ρ = -0.03, p = 0.88,  95% CI [-0.29, 0.22] |
| Left AV | partial ρ = -0.02, p = 0.93, 95% CI [-0.33, 0.34] | partial ρ = 0.1, p = 0.54,  95% CI [-0.27, 0.41] | partial ρ = 0.19, p = 0.24,  95% CI [-0.15, 0.56] | partial ρ = 0.26, p = 0.11,  95% CI [-0.11, 0.56] | partial ρ = 0.08, p = 0.62,  95% CI [-0.26, 0.41] |
| Right AV | partial ρ = -0.001, p = 0.99, 95% CI [-0.3, 0.35] | partial ρ = 0.15, p = 0.35,  95% CI [-0.16, 0.45] | partial ρ = -0.06, p = 0.71,  95% CI [-0.37, 0.26] | partial ρ = 0.007, p = 0.97,  95% CI [-0.35, 0.32] | partial ρ = 0.08, p = 0.63,  95% CI [-0.32, 0.43] |
| Left Pc | partial ρ = -0.00008, p = 1, 95% CI [-0.28, 0.3] | partial ρ = -0.18, p = 0.28,  95% CI [-0.43, 0.12] | partial ρ = -0.34, p = 0.03,  95% CI [-0.6, -0.02] | partial ρ = -0.07, p = 0.66,  95% CI [-0.38, 0.23] | partial ρ = -0.002, p = 0.99,  95% CI [-0.37, 0.36] |
| Right Pc | partial ρ = -0.21, p = 0.2, 95% CI [-0.51, 0.13] | partial ρ = 0.07, p = 0.69,  95% CI [-0.27, 0.33] | partial ρ = -0.28, p = 0.08,  95% CI [-0.55, 0.06] | partial ρ = -0.14, p = 0.4,  95% CI [-0.5, 0.2] | partial ρ = -0.04, p = 0.79,  95% CI [-0.33, 0.26] |
| Left VLp | partial ρ = -0.06, p = 0.72, 95% CI [-0.35, 0.25] | partial ρ = -0.27, p = 0.09,  95% CI [-0.52, 0.04] | partial ρ = -0.2, p = 0.22,  95% CI [-0.48, 0.09] | partial ρ = -0.21, p = 0.19,  95% CI [-0.5, 0.13] | partial ρ = -0.00006, p = 1,  95% CI [-0.33, 0.36] |
| Right VLp | partial ρ = 0.12, p = 0.47, 95% CI [-0.2, 0.47] | partial ρ = -0.12, p = 0.45,  95% CI [-0.44, 0.21] | partial ρ = -0.07, p = 0.65,  95% CI [-0.35, 0.26] | partial ρ = -0.15, p = 0.35,  95% CI [-0.51, 0.21] | partial ρ = 0.02, p = 0.88,  95% CI [-0.23, 0.3] |
| Left LP | partial ρ = -0.22, p = 0.18, 95% CI [-0.47, 0.05] | partial ρ = -0.18, p = 0.28,  95% CI [-0.45, 0.13] | partial ρ = 0.23, p = 0.15,  95% CI [-0.1, 0.54] | partial ρ = -0.22, p = 0.18,  95% CI [-0.42, 0.12] | partial ρ = -0.4, p = 0.01,  95% CI [-0.67, -0.07] |
| Right LP | partial ρ = -0.15, p = 0.36, 95% CI [-0.41, 0.13] | partial ρ = -0.29, p = 0.07,  95% CI [-0.56, 0.02] | partial ρ = 0.17, p = 0.29,  95% CI [-0.16, 0.51] | partial ρ = -0.29, p = 0.07,  95% CI [-0.55, 0.007] | partial ρ = -0.42, p = 0.007,  95% CI [-0.66, -0.16] |
| Left LD | partial ρ = -0.34, p = 0.035, 95% CI [-0.61, -0.04] | partial ρ = -0.3, p = 0.06,  95% CI [-0.61, 0.005] | partial ρ = -0.04, p = 0.8,  95% CI [-0.35, 0.31] | partial ρ = -0.21, p = 0.2,  95% CI [-0.51, 0.1] | partial ρ = -0.38, p = 0.02,  95% CI [-0.65, -0.08] |
| Right LD | partial ρ = -0.06, p = 0.74, 95% CI [-0.37, 0.26] | partial ρ = -0.07, p = 0.69,  95% CI [-0.38, 0.27] | partial ρ = 0.13, p = 0.44,  95% CI [-0.21, 0.45] | partial ρ = -0.09, p = 0.6,  95% CI [-0.37, 0.21] | partial ρ = -0.23, p = 0.15,  95% CI [-0.51, 0.09] |
| Left whole thalamus | partial ρ = -0.003, p = 0.99, 95% CI [-0.3, 0.29] | partial ρ = -0.36, p = 0.02,  95% CI [-0.59, -0.08] | partial ρ = -0.32, p = 0.04,  95% CI [-0.55, -0.04] | partial ρ = -0.33, p = 0.04,  95% CI [-0.59, 0.02] | partial ρ = -0.07, p = 0.65,  95% CI [-0.39, 0.25] |
| Right whole thalamus | partial ρ = 0.05, p = 0.75, 95% CI [-0.25, 0.41] | partial ρ = 0.01, p = 0.94,  95% CI [-0.32, 0.3] | partial ρ = -0.07, p = 0.68,  95% CI [-0.43, 0.25] | partial ρ = -0.08, p = 0.65,  95% CI [-0.46, 0.29] | partial ρ = 0.03, p = 0.87,  95% CI [-0.22, 0.26] |

Partial correlations between the ranked affective temperament scores and ranked thalamic nucleus volumes were calculated while controlling for ranked age, sex, and ranked eTIV.

Statistical significance was defined as a Bonferroni-corrected threshold of P < 0.00019. Boldface indicates statistically significant results.

**Table S4. Associations between affective temperament scores and thalamic nucleus volumes assessed using partial Spearman’s rank correlation coefficients adjusted** **for ranked age, sex, and ranked ipsilateral whole thalamus volume.**

Abbreviation; Anteroventral: AV, Central medial: CeM, Centromedian: CM, Central lateral: CL, Laterodorsal: LD, Lateral geniculate: LGN, Lateral posterior: LP, Limitans (suprageniculate): L-SG, Medial geniculate: MGN, Mediodorsal lateral parvocellular: MDl, Mediodorsal medial magnocellular: MDm, Paracentral: Pc, Parafascicular: Pf, Paratenial: Pt, Pulvinar anterior: PuA, Pulvinar inferior: PuI, Pulvinar lateral: PuL, Pulvinar medial: PuM, Reuniens (medial ventral): MV-Re, Ventral anterior: VA, Ventral anterior magnocellular: VAmc, Ventral lateral anterior: VLa, Ventral lateral posterior: VLp, Ventromedial: VM, Ventral posterolateral: VPL

|  | Depressive | Cyclothymic | Hyperthymic | Irritable | Anxious |
| --- | --- | --- | --- | --- | --- |
| Left LGN | partial ρ = 0.05, p = 0.78, 95% CI [-0.28, 0.38] | partial ρ = 0.05, p = 0.76,  95% CI [-0.29, 0.4] | partial ρ = 0.23, p = 0.15,  95% CI [-0.04, 0.49] | partial ρ = 0.006, p = 0.97,  95% CI [-0.27, 0.27] | partial ρ = -0.07, p = 0.66,  95% CI [-0.35, 0.24] |
| Right LGN | partial ρ = 0.06, p = 0.74  95% CI [-0.28, 0.38] | partial ρ = 0.04, p = 0.81,  95% CI [-0.27, 0.36] | partial ρ = 0.02, p = 0.89,  95% CI [-0.34, 0.32] | partial ρ = 0.23, p = 0.15,  95% CI [-0.07, 0.5] | partial ρ = 0.15, p = 0.35,  95% CI [-0.15, 0.42] |
| Left MGN | partial ρ = 0.13, p = 0.42, 95% CI [-0.17, 0.4] | partial ρ = 0.11, p = 0.52,  95% CI [-0.25, 0.43] | partial ρ = -0.17, p = 0.3,  95% CI [-0.47, 0.19] | partial ρ = 0.06, p = 0.73,  95% CI [-0.26, 0.37] | partial ρ = 0.18, p = 0.27,  95% CI [-0.16, 0.51] |
| Right MGN | partial ρ = 0.08, p = 0.63, 95% CI [-0.27, 0.4] | partial ρ = -0.03, p = 0.87,  95% CI [-0.34, 0.31] | partial ρ = -0.32, p = 0.045,  95% CI [-0.6, 0.04] | partial ρ = 0.009, p = 0.96,  95% CI [-0.34, 0.41] | partial ρ = 0.12, p = 0.46,  95% CI [-0.21, 0.43] |
| Left Pul | partial ρ = -0.23, p = 0.16,  95% CI [-0.53, 0.11] | partial ρ = -0.06, p = 0.72,  95% CI [-0.35, 0.26] | partial ρ = 0.04, p = 0.82,  95% CI [-0.3, 0.38] | partial ρ = 0.02, p = 0.92,  95% CI [-0.28, 0.33] | partial ρ = -0.26, p = 0.11,  95% CI [-0.55, 0.06] |
| Right Pul | partial ρ= -0.11, p = 0.51, 95% CI [-0.38, 0.25] | partial ρ = 0.32, p = 0.046,  95% CI [0.03, 0.57] | partial ρ = 0.17, p = 0.28,  95% CI [-0.14, 0.49] | partial ρ = 0.2, p = 0.21,  95% CI [-0.1, 0.46] | partial ρ = -0.09, p = 0.6,  95% CI [-0.39, 0.22] |
| Left PuM | partial ρ = -0.27, p = 0.1, 95% CI [-0.55, 0.06] | partial ρ = -0.07, p = 0.68,  95% CI [-0.37, 0.2] | partial ρ = -0.24, p = 0.14,  95% CI [-0.51, 0.08] | partial ρ = -0.28, p = 0.09,  95% CI [-0.59, 0.07] | partial ρ = -0.26, p = 0.11,  95% CI [-0.58, 0.08] |
| Right PuM | partial ρ = -0.22, p = 0.16, 95% CI [-0.49, 0.11] | partial ρ = 0.13, p = 0.44,  95% CI [-0.17, 0.37] | partial ρ = 0.05, p = 0.77,  95% CI [-0.28, 0.35] | partial ρ = 0.1, p = 0.53,  95% CI [-0.27, 0.5] | partial ρ = -0.17, p = 0.29,  95% CI [-0.46, 0.18] |
| Left L-Sg | partial ρ = -0.17, p = 0.3, 95% CI [-0.41, 0.07] | partial ρ = -0.25, p = 0.12,  95% CI [-0.52, 0.09] | partial ρ = -0.32, p = 0.048,  95% CI [-0.63, -0.01] | partial ρ = -0.05, p = 0.77,  95% CI [-0.33, 0.23] | partial ρ = 0.1, p = 0.53,  95% CI [-0.22, 0.42] |
| Right L-Sg | partial ρ = -0.12, p = 0.45, 95% CI [-0.42, 0.19] | partial ρ = -0.14, p = 0.4,  95% CI [-0.43, 0.16] | partial ρ = -0.52, p = 0.0007,  95% CI [-0.78, -0.18] | partial ρ = -0.03, p = 0.86,  95% CI [-0.36, 0.32] | partial ρ = 0.14, p = 0.41,  95% CI [-0.18, 0.42] |
| Left VPL | partial ρ = 0.24, p = 0.13,  95% CI [-0.08, 0.5] | partial ρ = -0.01, p = 0.93,  95% CI [-0.31, 0.26] | partial ρ = 0.36, p = 0.02,  95% CI [0.09, 0.58] | partial ρ = -0.05, p = 0.76,  95% CI [-0.3, 0.2] | partial ρ = 0.02, p = 0.91,  95% CI [-0.2, 0.23] |
| Right VPL | partial ρ = 0.12, p = 0.47,  95% CI [-0.16, 0.39] | partial ρ = -0.07, p = 0.69,  95% CI [-0.42, 0.3] | partial ρ = -0.02, p = 0.91,  95% CI [-0.27, 0.28] | partial ρ = 0.09, p = 0.59,  95% CI [-0.25, 0.43] | partial ρ = 0.1, p = 0.53,  95% CI [-0.23, 0.37] |
| Left CM | partial ρ = 0.06, p = 0.72, 95% CI [-0.25, 0.36] | partial ρ = -0.05, p = 0.75,  95% CI [-0.4, 0.32] | partial ρ = 0.14, p = 0.39,  95% CI [-0.17, 0.45] | partial ρ = 0.09, p = 0.59,  95% CI [-0.24, 0.4] | partial ρ = 0.09, p = 0.6,  95% CI [-0.25, 0.38] |
| Right CM | partial ρ = -0.001, p = 0.997,  95% CI [-0.32, 0.31] | partial ρ = -0.26, p = 0.11,  95% CI [-0.53, 0.02] | partial ρ = -0.12, p = 0.46,  95% CI [-0.38, 0.22] | partial ρ = 0.01, p = 0.93,  95% CI [-0.38, 0.3] | partial ρ = 0.07, p = 0.65,  95% CI [-0.24, 0.41] |
| Left VLa | partial ρ = -0.07, p = 0.66,  95% CI [-0.4, 0.24] | partial ρ = 0.06, p = 0.72,  95% CI [-0.27, 0.35] | partial ρ = 0.03, p = 0.84,  95% CI [-0.26, 0.28] | partial ρ = 0.46, p = 0.003,  95% CI [0.14, 0.7] | partial ρ = 0.33, p = 0.04,  95% CI [0.06, 0.53] |
| Right VLa | partial ρ = -0.05, p = 0.76, 95% CI [-0.36, 0.26] | partial ρ = -0.18, p = 0.27,  95% CI [-0.45, 0.11] | partial ρ = -0.19, p = 0.25,  95% CI [-0.43, 0.07] | partial ρ = -0.14, p = 0.37,  95% CI [-0.54, 0.2] | partial ρ = -0.07, p = 0.68,  95% CI [-0.43, 0.23] |
| Left PuA | partial ρ = 0.12, p = 0.48,  95% CI [-0.21, 0.42] | partial ρ = -0.1, p = 0.53,  95% CI [-0.39, 0.19] | partial ρ = -0.47, p = 0.002,  95% CI [-0.67, -0.18] | partial ρ = -0.28, p = 0.09,  95% CI [-0.56, 0.009] | partial ρ = 0.03, p = 0.86,  95% CI [-0.31, 0.36] |
| Right PuA | partial ρ = -0.05, p = 0.74, 95% CI [-0.38, 0.28] | partial ρ = 0.19, p = 0.25,  95% CI [-0.2, 0.48] | partial ρ = -0.1, p = 0.53,  95% CI [-0.38, 0.2] | partial ρ = -0.01, p = 0.94,  95% CI [-0.38, 0.37] | partial ρ = 0.02, p = 0.92,  95% CI [-0.32, 0.38] |
| Left MDm | partial ρ = 0.08, p = 0.65,  95% CI [-0.22, 0.45] | partial ρ = -0.11, p = 0.49,  95% CI [-0.4, 0.27] | partial ρ = -0.14, p = 0.38,  95% CI [-0.48, 0.15] | partial ρ = -0.29, p = 0.07,  95% CI [-0.54, 0.05] | partial ρ = -0.14, p = 0.39,  95% CI [-0.36, 0.13] |
| Right MDm | partial ρ = 0.007, p = 0.97, 95% CI [-0.29, 0.33] | partial ρ = -0.02, p = 0.93,  95% CI [-0.34, 0.32] | partial ρ = -0.31, p = 0.052,  95% CI [-0.56, -0.01] | partial ρ = -0.06, p = 0.7,  95% CI [-0.34, 0.24] | partial ρ = -0.24, p = 0.14,  95% CI [-0.51, 0.06] |
| Left Pf | partial ρ = -0.11, p = 0.49, 95% CI [-0.38, 0.15] | partial ρ = -0.23, p = 0.15,  95% CI [-0.54, 0.12] | partial ρ = 0.03, p = 0.83,  95% CI [-0.31, 0.38] | partial ρ = 0.06, p = 0.7,  95% CI [-0.27, 0.34] | partial ρ = 0.002, p = 0.99,  95% CI [-0.28, 0.3] |
| Right Pf | partial ρ = -0.01, p = 0.93, 95% CI [-0.32, 0.29] | partial ρ = -0.49, p = 0.001,  95% CI [-0.72, -0.23] | partial ρ = -0.36, p = 0.02,  95% CI [-0.62, -0.04] | partial ρ = -0.24, p = 0.14,  95% CI [-0.55, 0.07] | partial ρ = -0.01, p = 0.93,  95% CI [-0.3, 0.26] |
| Left VAmc | partial ρ = -0.11, p = 0.51, 95% CI [-0.43, 0.27] | partial ρ = -0.06, p = 0.72,  95% CI [-0.39, 0.26] | partial ρ = -0.03, p = 0.86,  95% CI [-0.34, 0.27] | partial ρ = 0.24, p = 0.14,  95% CI [-0.15, 0.54] | partial ρ = 0.03, p = 0.87,  95% CI [-0.37, 0.37] |
| Right VAmc | partial ρ = -0.26, p = 0.11, 95% CI [-0.58, 0.07] | partial ρ = -0.15, p = 0.35,  95% CI [-0.45, 0.14] | partial ρ = -0.22, p = 0.17,  95% CI [-0.52, 0.08] | partial ρ = -0.16, p = 0.34,  95% CI [-0.52, 0.14] | partial ρ = -0.16, p = 0.33,  95% CI [-0.51, 0.18] |
| Left MDl | partial ρ = -0.05, p = 0.78, 95% CI [-0.39, 0.33] | partial ρ = -0.22, p = 0.18,  95% CI [-0.47, 0.12] | partial ρ = -0.29, p = 0.07,  95% CI [-0.61, 0.007] | partial ρ = -0.35, p = 0.03,  95% CI [-0.62, 0.02] | partial ρ = -0.06, p = 0.72,  95% CI [-0.35, 0.3] |
| Right MDl | partial ρ = 0.09, p = 0.58, 95% CI [-0.21, 0.35] | partial ρ = 0.00009, p = 1,  95% CI [-0.31, 0.34] | partial ρ = -0.26, p = 0.1,  95% CI [-0.51, 0.04] | partial ρ = -0.11, p = 0.51,  95% CI [-0.38, 0.24] | partial ρ = 0.06, p = 0.72,  95% CI [-0.22, 0.33] |
| Left CeM | partial ρ = -0.17, p = 0.29, 95% CI [-0.48, 0.16] | partial ρ = 0.18, p = 0.27,  95% CI [-0.17, 0.51] | partial ρ = 0.1, p = 0.55,  95% CI [-0.2, 0.41] | partial ρ = 0.22, p = 0.17,  95% CI [-0.18, 0.54] | partial ρ = 0.004, p = 0.98,  95% CI [-0.36, 0.36] |
| Right CeM | partial ρ = -0.25, p = 0.12, 95% CI [-0.49, 0.07] | partial ρ = -0.31, p = 0.052,  95% CI [-0.56, -0.04] | partial ρ = -0.26, p = 0.11,  95% CI [-0.52, 0.04] | partial ρ = -0.27, p = 0.1,  95% CI [-0.55, 0.02] | partial ρ = -0.35, p = 0.03,  95% CI [-0.6, -0.04] |
| Left VA | partial ρ = -0.05, p = 0.74, 95% CI [-0.35, 0.26] | partial ρ = 0.08, p = 0.61,  95% CI [-0.22, 0.37] | partial ρ = 0.14, p = 0.4,  95% CI [-0.17, 0.41] | partial ρ = 0.41, p = 0.008,  95% CI [0.11, 0.66] | partial ρ = 0.19, p = 0.25,  95% CI [-0.07, 0.45] |
| Right VA | partial ρ = -0.22, p = 0.18, 95% CI [-0.49, 0.05] | partial ρ = -0.04, p = 0.82,  95% CI [-0.32, 0.29] | partial ρ = -0.36, p = 0.02,  95% CI [-0.61, -0.08] | partial ρ = -0.17, p = 0.28,  95% CI [-0.43, 0.12] | partial ρ = -0.11, p = 0.51,  95% CI [-0.41, 0.18] |
| Left MV(Re) | partial ρ = -0.32, p = 0.04, 95% CI [-0.6, 0.004] | partial ρ = -0.06, p = 0.74,  95% CI [-0.37, 0.34] | partial ρ = 0.05, p = 0.75,  95% CI [-0.24, 0.37] | partial ρ = 0.25, p = 0.11,  95% CI [-0.04, 0.5] | partial ρ = -0.14, p = 0.39,  95% CI [-0.45, 0.22] |
| Right MV(Re) | partial ρ = -0.24, p = 0.14, 95% CI [-0.52, 0.07] | partial ρ = -0.12, p = 0.47,  95% CI [-0.39, 0.14] | partial ρ = -0.14, p = 0.38,  95% CI [-0.43, 0.17] | partial ρ = -0.12, p = 0.47,  95% CI [-0.4, 0.18] | partial ρ = -0.27, p = 0.09,  95% CI [-0.58, -0.02] |
| Left VM | partial ρ = -0.06, p = 0.7, 95% CI [-0.36, 0.27] | partial ρ = -0.13, p = 0.43,  95% CI [-0.44, 0.17] | partial ρ = 0.02, p = 0.89,  95% CI [-0.26, 0.33] | partial ρ = 0.0003, p = 0.999,  95% CI [-0.28, 0.28] | partial ρ = 0.03, p = 0.84,  95% CI [-0.29, 0.32] |
| Right VM | partial ρ = -0.08, p = 0.61, 95% CI [-0.41, 0.19] | partial ρ = -0.08, p = 0.63,  95% CI [-0.41, 0.28] | partial ρ = -0.09, p = 0.59,  95% CI [-0.37, 0.26] | partial ρ = -0.13, p = 0.42,  95% CI [-0.5, 0.22] | partial ρ = 0.03, p = 0.85,  95% CI [-0.38, 0.33] |
| Left CL | partial ρ = -0.19, p = 0.25, 95% CI [-0.43, 0.1] | partial ρ = -0.05, p = 0.78,  95% CI [-0.34, 0.29] | partial ρ = 0.4, p = 0.01,  95% CI [0.2, 0.61] | partial ρ = 0.07, p = 0.68,  95% CI [-0.33, 0.39] | partial ρ = -0.32, p = 0.046,  95% CI [-0.6, -0.05] |
| Right CL | partial ρ = -0.18, p = 0.26, 95% CI [-0.45, 0.1] | partial ρ = -0.21, p = 0.18,  95% CI [-0.44, 0.11] | partial ρ = 0.08, p = 0.61,  95% CI [-0.22, 0.38] | partial ρ = -0.08, p = 0.65,  95% CI [-0.45, 0.27] | partial ρ = -0.25, p = 0.12,  95% CI [-0.52, 0.06] |
| Left PuL | partial ρ = 0.23, p = 0.16, 95% CI [-0.09, 0.52] | partial ρ = 0.11, p = 0.48,  95% CI [-0.16, 0.42] | partial ρ = -0.21, p = 0.19,  95% CI [-0.5, 0.11] | partial ρ = 0.0005, p = 0.998,  95% CI [-0.31, 0.31] | partial ρ = 0.24, p = 0.15,  95% CI [-0.09, 0.51] |
| Right PuL | partial ρ = 0.25, p = 0.12, 95% CI [-0.07, 0.57] | partial ρ = 0.42, p = 0.008,  95% CI [0.08, 0.72] | partial ρ = 0.14, p = 0.38,  95% CI [-0.18, 0.5] | partial ρ = 0.23, p = 0.15,  95% CI [-0.06, 0.51] | partial ρ = 0.35, p = 0.03,  95% CI [0.08, 0.6] |
| Left Pt | partial ρ = -0.18, p = 0.26, 95% CI [-0.56, 0.2] | partial ρ = -0.42, p = 0.007,  95% CI [-0.62, -0.17] | partial ρ = 0.02, p = 0.9,  95% CI [-0.27, 0.33] | partial ρ = -0.1, p = 0.56,  95% CI [-0.44, 0.22] | partial ρ = -0.18, p = 0.26,  95% CI [-0.54, 0.13] |
| Right Pt | partial ρ = -0.08, p = 0.64, 95% CI [-0.36, 0.2] | partial ρ = -0.17, p = 0.29,  95% CI [-0.5, 0.2] | partial ρ = -0.22, p = 0.18,  95% CI [-0.49, 0.13] | partial ρ = 0.13, p = 0.44,  95% CI [-0.23, 0.43] | partial ρ = -0.07, p = 0.65,  95% CI [-0.37, 0.22] |
| Left AV | partial ρ = -0.03, p = 0.88, 95% CI [-0.34, 0.31] | partial ρ = 0.18, p = 0.26,  95% CI [-0.15, 0.47] | partial ρ = 0.27, p = 0.09,  95% CI [-0.06, 0.57] | partial ρ = 0.39, p = 0.01,  95% CI [0.02, 0.66] | partial ρ = 0.1, p = 0.52,  95% CI [-0.25, 0.41] |
| Right AV | partial ρ = -0.04, p = 0.82, 95% CI [-0.4, 0.32] | partial ρ = 0.13, p = 0.42,  95% CI [-0.2, 0.46] | partial ρ = -0.05, p = 0.76,  95% CI [-0.35, 0.24] | partial ρ = 0.07, p = 0.69,  95% CI [-0.26, 0.38] | partial ρ = 0.07, p = 0.67,  95% CI [-0.31, 0.45] |
| Left Pc | partial ρ = -0.006, p = 0.97, 95% CI [-0.29, 0.29] | partial ρ = 0.06, p = 0.73,  95% CI [-0.26, 0.39] | partial ρ = -0.17, p = 0.28,  95% CI [-0.5, 0.17] | partial ρ = 0.21, p = 0.2,  95% CI [-0.16, 0.5] | partial ρ = 0.06, p = 0.72,  95% CI [-0.29, 0.39] |
| Right Pc | partial ρ = -0.26, p = 0.1, 95% CI [-0.52, 0.03] | partial ρ = 0.06, p = 0.7,  95% CI [-0.24, 0.31] | partial ρ = -0.28, p = 0.08,  95% CI [-0.53, 0.09] | partial ρ = -0.11, p = 0.5,  95% CI [-0.43, 0.16] | partial ρ = -0.06, p = 0.7,  95% CI [-0.37, 0.25] |
| Left VLp | partial ρ = -0.13, p = 0.44, 95% CI [-0.43, 0.2] | partial ρ = 0.13, p = 0.44,  95% CI [-0.16, 0.41] | partial ρ = 0.21, p = 0.2,  95% CI [-0.11, 0.49] | partial ρ = 0.25, p = 0.12,  95% CI [-0.09, 0.54] | partial ρ = 0.15, p = 0.36,  95% CI [-0.14, 0.4] |
| Right VLp | partial ρ = 0.11, p = 0.49, 95% CI [-0.21, 0.41] | partial ρ = -0.26, p = 0.11,  95% CI [-0.48, 0.06] | partial ρ = -0.05, p = 0.77,  95% CI [-0.29, 0.22] | partial ρ = -0.13, p = 0.43,  95% CI [-0.49, 0.25] | partial ρ = 0.001, p = 0.99,  95% CI [-0.38, 0.26] |
| Left LP | partial ρ = -0.23, p = 0.16, 95% CI [-0.5, 0.01] | partial ρ = -0.06, p = 0.72,  95% CI [-0.37, 0.25] | partial ρ = 0.38, p = 0.02,  95% CI [0.06, 0.64] | partial ρ = -0.1, p = 0.54,  95% CI [-0.45, 0.22] | partial ρ = -0.4, p = 0.01,  95% CI [-0.67, -0.11] |
| Right LP | partial ρ = -0.18, p = 0.26, 95% CI [-0.45, 0.11] | partial ρ = -0.31, p = 0.052,  95% CI [-0.54, 0.01] | partial ρ = 0.22, p = 0.16,  95% CI [-0.08, 0.54] | partial ρ = -0.29, p = 0.07,  95% CI [-0.61, 0.03] | partial ρ = -0.47, p = 0.002,  95% CI [-0.69, -0.22] |
| Left LD | partial ρ = -0.34, p = 0.03, 95% CI [-0.61, -0.05] | partial ρ = -0.28, p = 0.09,  95% CI [-0.57, 0.04] | partial ρ = -0.002, p = 0.99,  95% CI [-0.32, 0.34] | partial ρ = -0.16, p = 0.34,  95% CI [-0.45, 0.17] | partial ρ = -0.37, p = 0.02,  95% CI [-0.65, -0.05] |
| Right LD | partial ρ = -0.06, p = 0.7, 95% CI [-0.37, 0.23] | partial ρ = -0.07, p = 0.67,  95% CI [-0.39, 0.26] | partial ρ = 0.14, p = 0.41,  95% CI [-0.22, 0.47] | partial ρ = -0.08, p = 0.65,  95% CI [-0.36, 0.24] | partial ρ = -0.24, p = 0.14,  95% CI [-0.5, 0.1] |

Partial correlations between the ranked affective temperament scores and ranked thalamic nucleus volumes were calculated while controlling for ranked age, sex, and ranked ipsilateral whole thalamus volume.

# 2 Supplementary Figure

# Figure S1 The distributions of affective temperament scores

The Kolmogorov-Smirnov test revealed that none of the five affective temperament scores were normally distributed.

(A) Depressive temperament (p= 0.047), (B) Cyclothymic temperament (p= 0.0005), (C) Hyperthymic temperament (p= 0.009),

(D) Irritable temperament (p= 0.00002), (E) Anxious temperament (p= 0.00001).

# Figure S2 A representative example of the left thalamic paratenial nucleus identification based on FreeSurfer segmentation.

A representative example of the left thalamic paratenial nucleus (Pt) identification based on FreeSurfer segmentation in the present study. The purple region represents the left Pt.
